# Revision of the subgenus *Orthoscymnus* Canepari of *Scymnus* Kugelann (Coleoptera, Coccinellidae), with descriptions of four new species

**DOI:** 10.3897/zookeys.552.6167

**Published:** 2016-01-13

**Authors:** Xiaosheng Chen, Claudio Canepari, Xingmin Wang, Shunxiang Ren

**Affiliations:** 1 Engineering Research Center of Biological Control, Ministry of Education; College of Forestry and Landscape Architecture, South China Agricultural University, Guangzhou 510642, China; 2 Societa Entomologica Italiana Via Venezia 1, San Donato Milanese, Italy

**Keywords:** Taxonomy, Coccinelloidea, new species, new combination, Himalaya, China, Nepal

## Abstract

The subgenus *Orthoscymnus* Canepari, 1997 of *Scymnus* Kugelann, 1794 is herein revised. Seven species of the *Orthoscymnus* fauna are recognized, of which four species, Scymnus (Orthoscymnus) jilongicus **sp. n.**, Scymnus (Orthoscymnus) paradoxus **sp. n.**, Scymnus (Orthoscymnus) crispatus **sp. n.** and Scymnus (Orthoscymnus) duomaculatus **sp. n.**, are described as new to science. Scymnus (Orthoscymnus) rhododendri Canepari is recorded from China for the first time. Scymnus (Pullus) robustibasalis Yu is transferred to the subgenus *Orthoscymnus* (**comb. n.**). All species are diagnosed, described and illustrated, and distributions are provided for each species. A key to the species is included.

## Introduction

Most members of the family Coccinellidae are important natural enemies of pest, such as whiteflies, aphids, mealybugs, scales and mites, and playing an important role in regulating their populations. Recently, this family was classified in the superfamily Coccinelloidea along with eight other families (Robertson et al. 2015).

The genus *Scymnus* Kugelann, 1794 comprises eight subgenera and more than 800 species distributed worldwide (Chen et al. 2013, 2015). In the modern classification, *Scymnus* has been placed within the tribe Scymnini Mulsant, 1846 in the subfamily Scymninae (Sasaji 1968; Kovář 1996). However, Ślipiński (2007) proposed only two subfamilies, Microweiseinae and Coccinellinae, for the family Coccinellidae, *Scymnus* was classified in the tribe Coccidulini of the broadly defined subfamily Coccinellinae. This classification was supported by Giorgi et al. (2009) and Seago et al. (2011) based on molecular and morphological studies.

The subgenus *Orthoscymnus* Canepari, 1997 of *Scymnus* Kugelann, 1794 was established for two new species from Nepal, Scymnus (Orthoscymnus) smetanai and Scymnus (Orthoscymnus) rhododendri (Canepari, 1997). So far, this subgenus only occurred in the Himalaya region and included two species.

In the present paper, seven species of the subgenus *Orthoscymnus* are recognized, including four new species described here. Scymnus (Orthoscymnus) rhododendri Canepari, 1997 is newly recorded from China. Scymnus (Pullus) robustibasalis Yu, 2000 is transferred into the subgenus *Orthoscymnus* based on the characters of the male genitalia, particularly the robust penis capsule. Diagnoses, detailed descriptions and illustrations are provided for each species.

### Materials and methods

The morphological terms follow Ślipiński (2007) and Ślipiński and Tomaszewska (2010). Depositories of the type materials are abbreviated as follows:

SCAU
South China Agricultural University, Guangzhou, China;

BAAF
 Beijing Academy of Agricultural and Forestry Science, Beijing, China;

MNHG
Museum National d’Histoire Naturelle, Genéve;

SMNS
Staatliches Museum für Naturkunde, Stuttgart, Germany.

Measurements were made using a micrometer attached to a SteREO Discovery V20 dissecting stereoscope and are defined as follows: (TL) total length, from apical margin of clypeus to apex of elytra; (TW) total width, across both elytra at widest part; (TH) total height, at highest part of elytra; (HW) head width, at widest part including eyes; (PL) pronotal length, from the middle of anterior margin to the base of pronotum; (PW) pronotal width at widest part; (EL) elytral length, along suture from base to apex including scutellum; (EW) elytral width, equal to TW.

Male genitalia were dissected, cleared in a 10% solution of NaOH, and placed on slides for further study. Illustrations of morphological details were made from slide preparations using a camera (Coolsnap-Procf & CRI Micro*Color) attached to an Olympus BX51 compound microscope. After examination, they were transferred to a small card covered with neutral balsam and pinned beneath the specimen.

Photographs of the whole beetles were executed using digital cameras (AxioCam HRc) and composite images generated with AXIO VISION REL. 4.8 softwares. The final plates were prepared using ADOBE PHOTOSHOP CS 8.0.

## Taxonomy

### Genus *Scymnus* Kugelann, 1794

#### Subgenus Orthoscymnus

Taxon classificationAnimaliaColeopteraCoccinellidae

Canepari, 1997

Orthoscymnus Canepari, 1997: 16. Type species: Scymnus (Orthoscymnus) smetanai Canepari, 1997, by original designation.

##### Diagnosis.

Body compact, round oval or elongate oval, slightly convex, dorsum densely pubescent. Head small, frons finely punctate. Eyes finely faceted. Antennae composed of 11 antennomeres. Antennal club compact, composed of 3 antennomeres. Clypeus transverse with anterior margin straight. Labrum transverse, entirely exposed. Mandible bifid apically. Pronotum moderately convex. Prosternum T-shaped. Prosternal process bearing distinct lateral carinae, convergent and extending to anterior margin. Abdomen with six ventrites. Abdominal postcoxal lines recurved and complete laterally. Tarsi with 4 tarsomeres; claws bifid, each with sharp basal tooth. Male genitalia with penis guide symmetrical. Penis stout with an irregular basal capsule, usually highly sclerotized. Female genitalia with sub-horizontal coxites, infundibulum elongate, spermatheca worm-shaped.

##### Remarks.

Orthoscymnus shares many characters with subgenus Pullus Mulsant, such as antennae composed of 11 antennomeres and the complete abdominal postcoxal lines, but can be distinguished from the latter by the female genitalia with sub-horizontal coxites (see Canepari 1997). In the subgenus *Pullus*, coxites are elongate, triangular.

##### Key to the species of the subgenus *Orthoscymnus*

**Table:** 

1	Elytra black; apex of penis without thread-like appendage	**2**
–	Elytra entirely reddish brown (Fig. 1a); apex of penis with short thread-like appendage (Fig. 1f); length 1.49–1.59 mm	**Scymnus (Orthoscymnus) smetanai Canepari**
2	Head and pronotum brown; penis capsule with short inner arm and large outer arm	**3**
–	Head and pronotum black; penis capsule with both arms well developed	**6**
3	Elytra with apical margin narrowly brown	**4**
–	Elytra with large X-shaped yellow area extending from basal 1/4 to elytral apex (Fig. 2a); length 1.96–2.06mm	**Scymnus (Orthoscymnus) jilongicus Chen & Ren, sp. n.**
4	Parameres shorter than penis guide in lateral view; penis guide not spade-shaped in ventral view	**5**
–	Parameres longer than penis guide in lateral view (Fig. 3h); penis guide extremely broad, spade-shaped in ventral view (Fig. 2a); length 1.64–1.96 mm	**Scymnus (Orthoscymnus) paradoxus Chen & Ren, sp. n.**
5	Penis guide sub-rectangular through 3/4 of its length in ventral view (Fig. 4g); parameres narrow at base and expanded toward their apices in lateral view (Fig. 4h); length 1.60–1.67 mm	**Scymnus (Orthoscymnus) rhododendri Canepari**
–	Penis guide sub-triangular in ventral view (Fig. 5g); parameres regularly elongate oval in lateral view (Fig. 5h); length 1.59–1.65 mm	**Scymnus (Orthoscymnus) crispatus Chen & Ren, sp. n.**
6	Elytra without brown spot (Fig. 6a); parameres distinctly shorter than penis guide in lateral view (Fig. 6h); length 1.60–1.67 mm	**Scymnus (Orthoscymnus) robustibasalis Yu**
–	Elytra with two kidney-shaped brown spots near suture (Fig. 7a); parameres slightly longer than penis guide in lateral view (Fig. 7h); length 1.62–1.83 mm	**Scymnus (Orthoscymnus) duomaculatus Chen & Ren, sp. n.**

**Figure 1.**
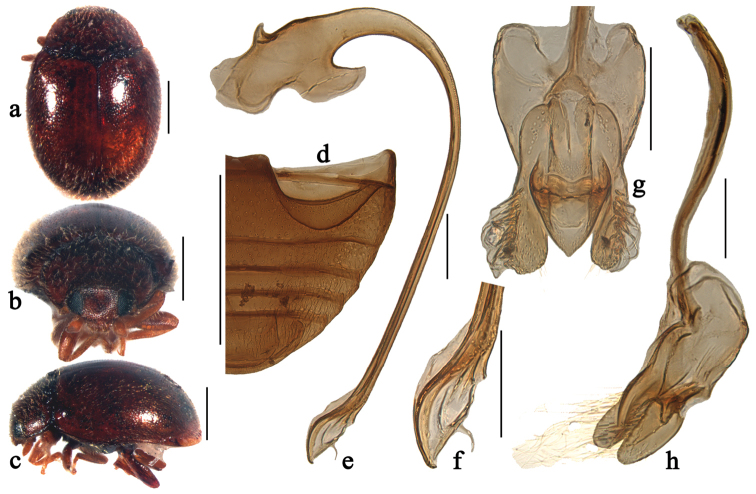
Scymnus (Orthoscymnus) smetanai Canepari, 1997: **a** dorsal view **b** frontal view **c** lateral view **d** abdomen **e** penis **f** apex of penis **g** tegmen, ventral view **h** tegmen, lateral view. Scale bars: **a–d**: 0.5 mm, **e–h**: 0.1 mm.

**Figure 2.**
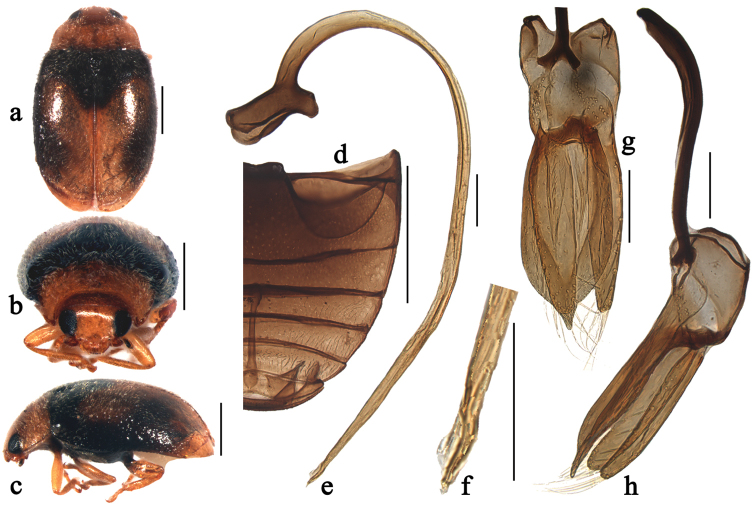
Scymnus (Orthoscymnus) jilongicus Chen & Ren, sp. n.: **a** dorsal view **b** frontal view **c** lateral view **d** abdomen; **e** penis **f** apex of penis **g** tegmen, ventral view **h** tegmen, lateral view. Scale bars: **a–d**: 0.5 mm, **e–h**: 0.1 mm.

**Figure 3.**
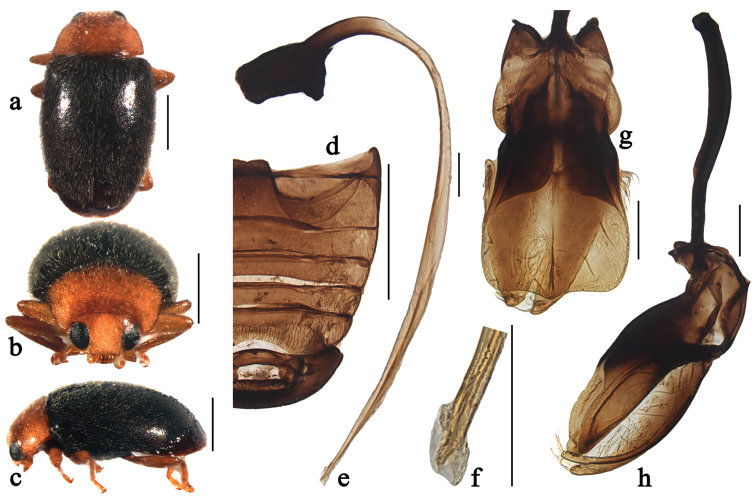
Scymnus (Orthoscymnus) paradoxus Chen & Ren, sp. n.: **a** dorsal view **b** frontal view **c** lateral view **d** abdomen **e** penis **f** apex of penis **g** tegmen, ventral view **h** tegmen, lateral view. Scale bars: **a–d**: 0.5 mm, **e–h**: 0.1 mm.

**Figure 4.**
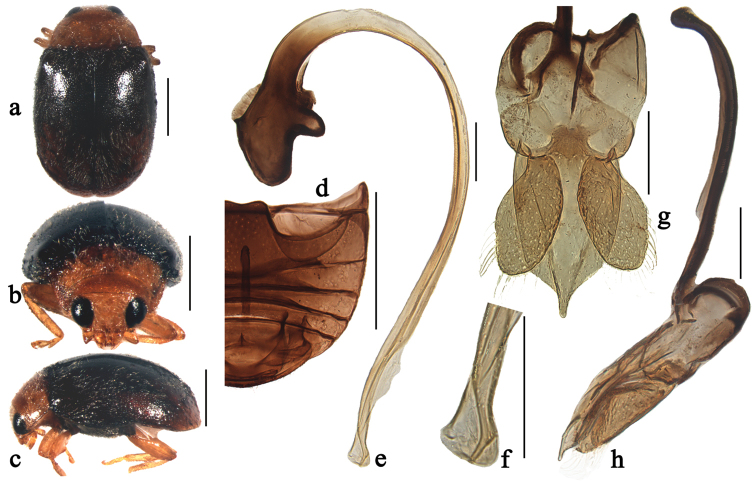
Scymnus (Orthoscymnus) rhododendri Canepari, 1997: **a** dorsal view **b** frontal view **c** lateral view **d** abdomen **e** penis **f** apex of penis **g** tegmen, ventral view **h** tegmen, lateral view. Scale bars: **a–d**: 0.5 mm, **e–h**: 0.1 mm.

**Figure 5.**
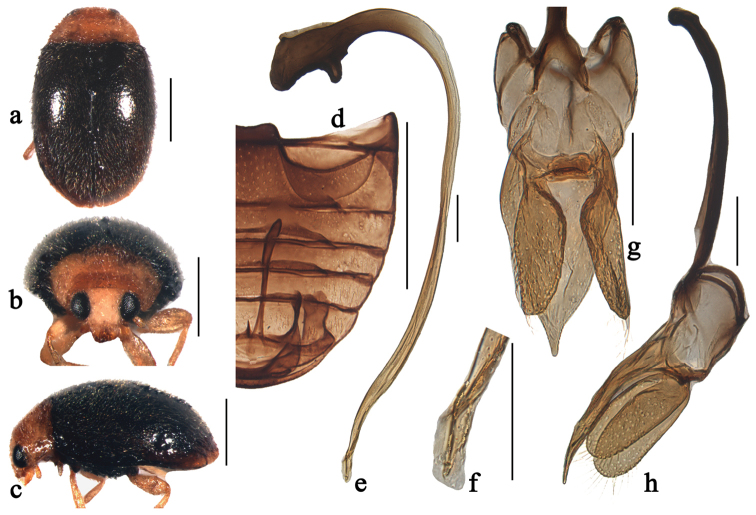
Scymnus (Orthoscymnus) crispatus Chen & Ren, sp. n.: **a** dorsal view **b** frontal view **c** lateral view **d** abdomen **e** penis **f** apex of penis **g** tegmen, ventral view **h** tegmen, lateral view. Scale bars: **a–d**: 0.5 mm, **e–h**: 0.1 mm.

**Figure 6.**
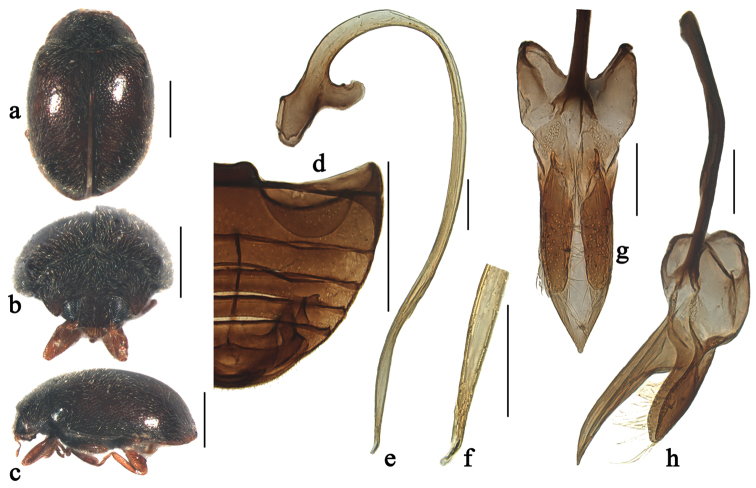
Scymnus (Orthoscymnus) robustibasalis (Yu, 2000), comb. n.: **a** dorsal view **b** frontal view **c** lateral view **d** abdomen **e** penis **f** apex of penis **g** tegmen, ventral view **h** tegmen, lateral view. Scale bars: **a–d**: 0.5 mm, **e–h**: 0.1 mm.

**Figure 7.**
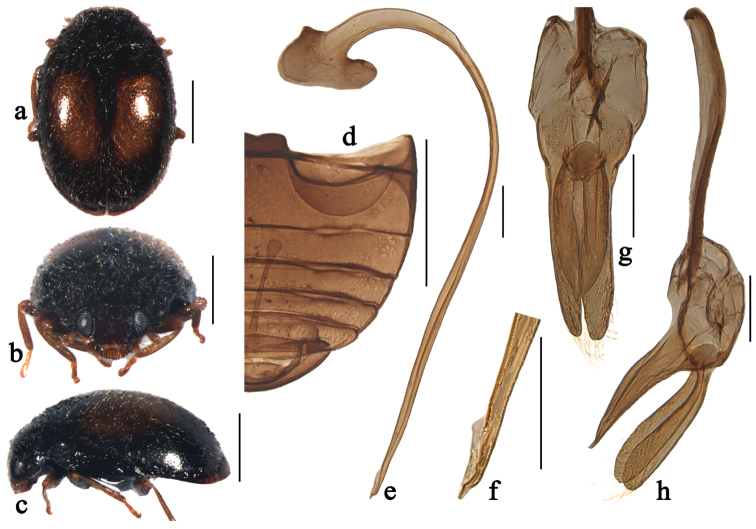
Scymnus (Orthoscymnus) duomaculatus Chen & Ren, sp. n.: **a** dorsal view **b** frontal view **c** lateral view **d** abdomen; **e** penis **f** apex of penis; **g** tegmen, ventral view **h** tegmen, lateral view. Scale bars: **a–d**: 0.5 mm, **e–h**: 0.1 mm.

#### Scymnus (Orthoscymnus) smetanai

Taxon classificationAnimaliaColeopteraCoccinellidae

Canepari, 1997

Figs 1a–h , 8

Scymnus (Orthoscymnus) smetanai Canepari, 1997: 17; Poorani 2002: 357; Kovář 2007: 586.

##### Diagnosis.

This species can be easily separated from other members of the subgenus *Orthoscymnus* by having entirely reddish brown body. It is also similar to Scymnus (Pullus) martensi Canepari in general appearance and particularly in the shape of abdominal postcoxal lines, but can be distinguished from it by the much smaller body and the swollen apex of penis bearing short thread-like appendage (Fig. 1f).

##### Description.

TL: 1.49–1.59 mm, TW: 1.09–1.19 mm, TH: 0.69–0.80 mm, TL/TW: 1.25–1.46, PL/PW: 0.56–0.64, EL/EW: 1.03–1.17, HW/PW: 0.54–0.59, PW/EW: 0.66–0.74.

Body rounded oval, moderately convex, entirely reddish brown, dorsum covered with white pubescence (Figs 1a–c).

Head with fine frontal punctures, slightly larger than eye facets, 1.0–2.0 diameters apart. Eyes densely faceted, interocular distance 0.45 times head width. Pronotal punctures as large as those on frons, 1.0–2.0 diameters apart. Surface of elytra with punctures larger than those on pronotum, separated by 2.0–3.0 diameters. Prosternal process trapezoidal, 2 times as long as its width at base; with lateral carinae extending to anterior margin of prosternum, distinctly convergent anteriorly. Abdominal postcoxal lines reaching 3/4 length of abdominal ventrite 1 (Fig. 1d), area enclosed by lines coarsely punctate, narrowly smooth along line. Abdominal ventrite 5 with apex truncate in male.

Male genitalia. Penis stout; penis capsule with small and curved inner arm, outer arm large bearing horn-shaped appendage (Fig. 1e); apex of penis distinctly swollen, bearing short thread-like appendage (Fig. 1f). Tegmen stout (Fig. 1g–h) with penis guide widest at base, gradually tapering to blunt apex in ventral view (Fig. 1g). Parameres longer than penis guide, densely covered with long setae at apices and inner sides (Fig. 1h).

Female externally similar to male but with abdominal ventrite 5 rounded apically.

##### Type material.

**Holotype**: male, “Nepal, Khandbari Distr., forest above Ahale, (27°27.62'N, 87°11.49'E), 2300m, 26. III. 82, leg. Smetana” (MNHG).

##### Other material examined.

**Nepal: Koshi**: 1♂, on the way from Dharan to Dhankuta, 26°52.94'N, 87°19.74'E, 400-700 m, 20. X. 2011, Chen XS leg.

##### Distribution.

Nepal (Koshi).

**Figure 8.**
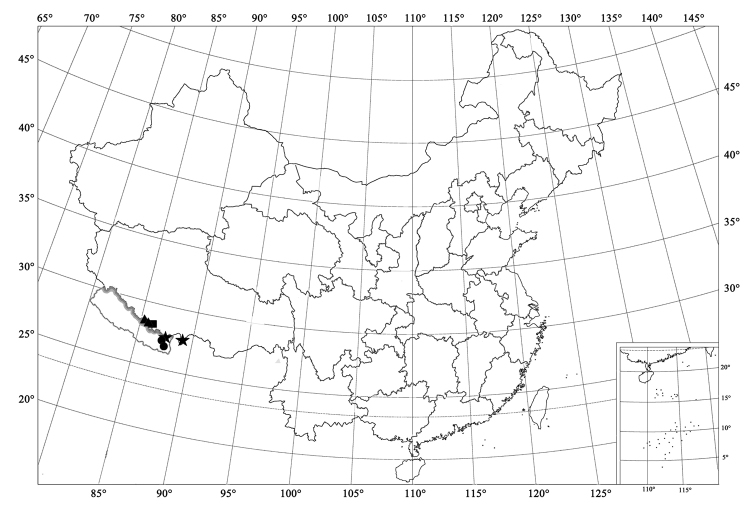
Distribution map. Scymnus (Orthoscymnus) smetanai Canepari (●); Scymnus (Orthoscymnus) jilongicus Chen & Ren, sp. n. (▲); Scymnus (Orthoscymnus) paradoxus Chen & Ren, sp. n. (■); Scymnus (Orthoscymnus) rhododendri Canepari (★).

#### Scymnus (Orthoscymnus) jilongicus

Taxon classificationAnimaliaColeopteraCoccinellidae

Chen & Ren sp. n.

http://zoobank.org/9BFB1A7E-BDE1-4F86-83E2-1E0592F0490D

Figs 2a–h , 8

##### Diagnosis.

This species can be separated from other species within the subgenus *Orthoscymnus* by having a large X-shaped yellow macula on elytra, extending from basal fourth to elytral apex (Fig. 2a). It is also similar to Scymnus (Pullus) testacecollis Kapur in male genitalia, but can be distinguished from it by having slender parameres slightly shorter than penis guide in lateral view (Fig. 2h). In Scymnus (Pullus) testacecollis, the elytra is black with apical 1/4 testaceous; the parameres are narrow at base and expanding gradually toward apex, nearly as long as penis guide in lateral view (see Kapur 1963).

##### Description.

TL: 1.96–2.06mm, TW: 1.15–1.25mm, TH: 0.81–0.82mm, TL/TW: 1.65–1.70, PL/PW: 0.53–0.55, EL/EW: 1.27–1.34, HW/PW: 0.59–0.61, PW/EW: 0.74–0.76.

Body elongate oval, slightly convex, dorsum covered with white pubescence (Fig. 2a–c). Head, antennae and mouthparts yellowish brown. Pronotum yellow. Scutellum black. Elytra black with large X-shaped, yellow macula at middle, extending to its apex (Fig. 4a). Prothoracic hypomeron yellow. Prosternum brown to black. Mesoventrite and metaventrite black. Elytral epipleuron brown with inner and outer margins black. Legs yellowish brown.

Head with fine frontal punctures, as large as eye facets, 1.0–2.0 diameters apart. Eyes densely faceted, interocular distance 0.51 times head width. Pronotal punctures similar to those on frons, 1.0–1.5 diameters apart. Surface of elytra with punctures much coarser than those on pronotum, separated by 1.0–2.0 diameters. Prosternal process rectangular, 3.5 times as long as its width at base; with lateral carinae parallel, extending to anterior margin of prosternum. Abdominal postcoxal lines reaching 2/3 length of abdominal ventrite 1 (Fig. 2d), area enclosed by lines coarsely punctate, narrowly smooth along line. Abdominal ventrite 5 in male with apical margin shallowly emarginate and ventrite 6 strongly emarginate medially.

Male genitalia. Penis stout and long (Fig. 2e); penis capsule highly sclerotized with short inner arm and large outer arm; apex of penis with membranous appendage (Fig. 2f). Tegmen extremely stout (Figs 2g–h) with penis guide parallel-sided from base to apical 3/4 length, then tapering gradually to pointed apex in ventral view (Fig. 2g), flattened and nearly straight in lateral view (Fig. 2h). Parameres narrow, slightly shorter than penis guide, densely covered with long setae at apices (Fig. 2h).

Female externally similar to male but with apex of abdominal ventrite 5 truncate and ventrite 6 rounded apically.

##### Type material.

**Holotype**: male, No. SCAU (E) 13196, **China: Tibet**: Jilong Town, Jilong County, 28°23.00'N, 85°19.60'E, ca 2900 m, 29. X. 2011, Huo LZ leg. **Paratypes (17)**: 2♂13♀ with same data as holotype. 2♂, Zhangmu Port, Nielamu, Rikaze, 27°58.47'N, 85°58.15'E, ca 3000 m, 28. IX. 2009, Chen XS leg. (SCAU)

##### Etymology.

The species name is derived from the type locality, Jilong Town, Tibet.

#### Scymnus (Orthoscymnus) paradoxus

Taxon classificationAnimaliaColeopteraCoccinellidae

Chen & Ren sp. n.

http://zoobank.org/3FF40A9D-ED14-4140-9923-7B9FB0989248

Figs 3a–h , 8

##### Diagnosis.

This species can be easily recognized by its elongate and compressed body and the peculiar characters on male genitalia, particularly the extremely broad, spade-shaped penis guide in ventral view (Fig. 3g).

##### Description.

TL: 1.64–1.96 mm, TW: 0.97–1.09 mm, TH: 0.70–0.77 mm, TL/TW: 1.69–1.79, PL/PW: 0.55–0.56, EL/EW: 1.29–1.37, HW/PW: 0.59–0.61, PW/EW: 0.74–0.79.

Body elongate oval, slightly convex, dorsum covered with white pubescence (Figs 3a–c). Head, antennae and mouthparts yellowish brown. Pronotum yellow. Scutellum black. Elytra black with apical margin narrowly brown. Prothoracic hypomeron and prosternum yellow. Mesoventrite, metaventrite and elytral epipleura black. Legs yellowish brown.

Head with fine frontal punctures, as large as eye facets, 0.5–1.0 diameter apart. Eyes densely faceted, interocular distance 0.47 times head width. Pronotal punctures larger than those on frons, 1.0–2.0 diameters apart. Surface of elytra with punctures much larger than those on pronotum, separated by 2.0–3.0 diameters. Prosternal process rectangular, 5 times as long as its width at base; with lateral carinae parallel, extending to anterior margin of prosternum. Abdominal postcoxal lines reaching 4/5 length of abdominal ventrite 1 (Fig. 3d), area enclosed by lines finely punctate, broadly smooth along line. Abdominal ventrites 5 and 6 in male strongly emarginate apically.

Male genitalia. Penis slender (Fig. 3e); penis capsule highly sclerotized with short inner arm and large outer arm; apex of penis with membranous appendage (Fig. 3f). Tegmen extremely stout (Fig. 3g–h) with penis guide broad, spade-shaped in ventral view (Fig. 3g). Parameres very narrow in lateral view, slightly longer than penis guide, sparsely covered with long setae at apices and inner sides (Fig. 3h).

Female externally similar to male but with black pronotum, abdominal ventrite 5 truncate and ventrite 6 rounded apically.

##### Type material.

**Holotype**: male, No. SCAU (E) 13193, **China: Tibet**: Zhangmu Town, Nielamu County, 27°58.47'N, 85°58.15'E, ca 2200 m, 31. X. 2011, Chen XS leg. **Paratypes (5)**: 5♀ with same data as holotype. (SCAU)

##### Distribution.

China (Tibet).

##### Etymology.

The species name is an adjective derived from Latin (‘*paradoxus*’ = strange), referring to its peculiar shape of penis guide.

#### Scymnus (Orthoscymnus) rhododendri

Taxon classificationAnimaliaColeopteraCoccinellidae

Canepari, 1997

Figs 4a–h , 8

Scymnus (Orthoscymnus) rhododendri Canepari, 1997: 17; Poorani 2002: 357; Kovář 2007: 584.

##### Diagnosis.

This species is similar to Scymnus (Orthoscymnus) crispatus sp. n. in general appearance and male genitalia, but can be separated from it by having swollen apex of penis (Fig. 4f), extremely broad penis guide in ventral view (Fig. 4g) and the parameres distinctly constricted at base, then expanding toward apex in ventral view (Fig. 4h).

##### Description.

TL: 1.60–1.67 mm, TW: 1.05–1.08 mm, TH: 0.75–0.78 mm, TL/TW: 1.52–1.55, PL/PW: 0.49–0.53, EL/EW: 1.15–1.17, HW/PW: 0.61–0.62, PW/EW: 0.73–0.75.

Body elongate oval, moderately convex, dorsum covered with white pubescence (Figs 4a–c). Head, antennae and mouthparts yellowish brown. Pronotum brown, sometimes with black marking at base. Scutellum black. Elytra black with apical margin narrowly brown. Prothoracic hypomeron and prosternum yellowish brown. Mesoventrite, metaventrite and elytral epipleura black. Legs yellowish brown.

Head with fine frontal punctures, as large as eye facets, 1.0–2.0 diameters apart. Eyes densely faceted, interocular distance 0.42 times head width. Pronotal punctures larger than those on frons, 1.0–2.0 diameters apart. Surface of elytra with punctures much coarser than those on pronotum, separated by 2.0–3.0 diameters. Prosternal process trapezoidal, 2 times as long as its width at base; with lateral carinae extending to anterior margin of prosternum, distinctly convergent anteriorly. Abdominal postcoxal lines extending nearly to posterior margin of abdominal ventrite 1 (Fig. 4d), area enclosed by lines finely punctate, broadly smooth along line. Abdominal ventrites 5 and 6 in male strongly emarginate apically.

Male genitalia. Penis stout (Fig. 4e); penis capsule highly sclerotized with tiny inner arm and large outer arm; apex of penis distinctly swollen with membranous appendage at apical 1/5 length (Fig. 4f). Tegmen stout (Figs 4g–h) with penis guide wide, with sides subparallel from base to its apical 3/4 length, then tapering gradually to pointed apex in ventral view (Fig. 4g), and its apex slightly curved outwardly in lateral view (Fig. 4h). Parameres constricted at base with expanded apex, distinctly shorter than penis guide, sparsely covered with long setae at apices (Fig. 4h).

Female unknown.

##### Type material.

**Holotype**: male, “Nepal, Sankhua Sabha Distr., above Pahakhola, (27°39.40'N, 87°16.12'E), *Quercus semecarpifolia*-Rhododendron, 2600–2800 m, 3. VI. 1988, leg Martens & Schawaller” (SMNS).

##### Other material examined.

**China: Tibet**: 2♂, Xiayadong Village, Yadong County, 28°29.29'N, 97°1.36'E, ca 2800 m, 1. X. 2009, Chen XS leg. 5♂, Xiayadong Village, Yadong County, 28°29.29'N, 97°1.36'E, ca 2800 m, 29–30. IX. 2009, Chen XS leg.

##### Distribution.

China (Tibet) new distribution; Nepal.

#### Scymnus (Orthoscymnus) crispatus

Taxon classificationAnimaliaColeopteraCoccinellidae

Chen & Ren sp. n.

http://zoobank.org/CC4D9926-B36D-449E-AFB9-9FBD4CBD5F2E

Figs 5a–h , 9

##### Diagnosis.

This species is similar to Scymnus (Orthoscymnus) rhododendri in male genitalia, but can be distinguished from it by the elongate oval parameres (Fig. 5h) and the narrow, sub-triangular penis guide in ventral view (Fig. 5g).

##### Description.

TL: 1.59–1.65 mm, TW: 0.90–1.01 mm, TH: 0.68–0.76 mm, TL/TW: 1.59–1.63, PL/PW: 0.53–0.55, EL/EW: 1.27–1.32, HW/PW: 0.59–0.63, PW/EW: 0.73–0.74.

Body elongate oval, moderately convex, dorsum covered with white pubescence (Fig. 5a–c). Head yellow. Antennae and mouthparts yellowish brown. Pronotum yellow. Scutellum black. Elytra black with apical margin narrowly brown. Prothoracic hypomeron yellow. Prosternum brown. Mesoventrite, metaventrite and elytral epipleura black. Legs yellowish brown.

Head with fine frontal punctures, as large as eye facets, 0.5–1.0 diameter apart. Eyes densely faceted, interocular distance 0.43 times head width. Pronotal punctures slightly larger than those on frons, 1.0–2.0 diameters apart. Surface of elytra with punctures much larger than those on pronotum, separated by 2.0–3.0 diameters. Prosternal process trapezoidal, 4 times as long as its width at base; with lateral carinae extending to anterior margin of prosternum, distinctly convergent anteriorly. Abdominal postcoxal lines extending nearly to posterior margin of abdominal ventrite 1 (Fig. 5d), area enclosed by lines finely punctate, broadly smooth along line. Abdominal ventrite 5 strongly emarginate and ventrite 6 weakly emarginate apically in male.

Male genitalia. Penis stout (Fig. 5e); penis capsule highly sclerotized with tiny inner arm and large outer arm; apex of penis with membranous appendage (Fig. 5f). Tegmen stout (Fig. 5g–h) with penis guide slightly constricted at base, widest at basal 1/4 length, then tapering gradually to pointed apex in ventral view (Fig. 5g) and its apex curved outwardly in lateral view (Fig. 5h). Parameres elongate oval, shorter than penis guide, sparsely setose at apices (Fig. 5h).

Female externally similar to male but with abdominal ventrites 5 and 6 truncate apically. In some specimens, pronotum and elytra are entirely reddish brown.

##### Type material.

**Holotype**: male, No. SCAU (E) 13195, **China: Tibet**: Jilong Town, Jilong County, 28°23.00'N, 85°19.60'E, ca 2900 m, 29. X. 2011, Li WJ leg. **Paratypes (71)**: 15♂55♀ with same data as holotype. 1♂, Lebu Village, Cuona County, 27°48.63'N, 91°44.98'E, ca 2400 m, 24. X. 2011, Huo LZ leg. (SCAU)

##### Distribution.

China (Tibet).

**Figure 9.**
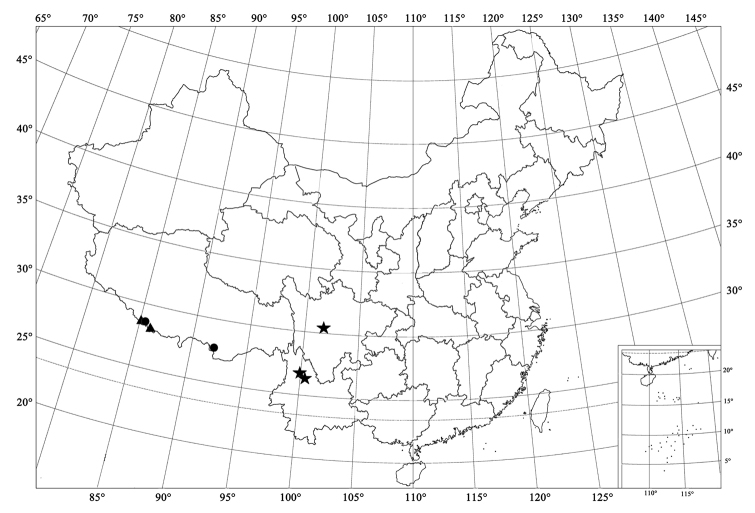
Distribution map. Scymnus (Orthoscymnus) crispatus Chen & Ren, sp. n. (●); Scymnus (Orthoscymnus) robustibasalis (Yu), comb. n. (★); Scymnus (Orthoscymnus) duomaculatus Chen & Ren, sp. n. (▲).

##### Etymology.

The species name is an adjective derived from Latin (‘*crispatus*’ = crispate), referring to its parameres with crispate surface in ventral view.

#### Scymnus (Orthoscymnus) robustibasalis

Taxon classificationAnimaliaColeopteraCoccinellidae

(Yu, 2000) comb. n.

Figs 6a–h , 9

Scymnus (Pullus) robustibasalis Yu in Yu et al. 2000: 180; Pang et al. 2004: 80; Kovář 2007: 587.

##### Diagnosis.

This species is most similar to Scymnus (Orthoscymnus) duomaculatus sp. n. in having black pronotum but can be easily separated from it by the black elytra (Fig. 6a) and parameres tapering toward apex, distinctly shorter than penis guide in lateral view (Fig. 6h).

##### Description.

TL: 1.69–1.76mm, TW: 1.10–1.21mm, TH: 0.76–0.83mm, TL/TW: 1.45–1.54, PL/PW: 0.47–0.52, EL/EW: 1.24–1.26, HW/PW: 0.56–0.57, PW/EW: 0.73–0.75.

Body elongate oval, moderately convex, dorsum covered with white pubescence (Figs 6a–c). Head, antennae and mouthparts dark brown. Pronotum black with anterior angles dark brown. Scutellum black. Elytra black with apical margin narrowly reddish brown. Prothoracic hypomeron dark brown. Prosternum, mesoventrite, metaventrite and elytral epipleura black. Legs yellowish brown.

Head with dense frontal punctures, slightly smaller than eye facets, 0.5–1.0 diameter apart. Eyes densely faceted, interocular distance 0.50 times head width. Pronotal punctures as large as those on frons, 1.0–1.5 diameters apart. Surface of elytra with punctures much larger than those on pronotum, separated by 1.0–2.0 diameters. Prosternal process trapezoidal, 2 times as long as its width at base; with lateral carinae extending to anterior margin of prosternum, slightly convergent anteriorly. Abdominal postcoxal lines reaching 3/4 length of abdominal ventrite 1 (Fig. 6d), area enclosed by lines finely punctate, broadly smooth along line. Abdominal ventrites 5 and 6 in male strongly emarginate apically.

Male genitalia. Penis robust and short, unevenly curved (Fig. 6e); penis capsule sclerotized with both arms well developed; apex of penis simple (Fig. 6f). Tegmen stout with tegminal strut black, highly sclerotized (Fig. 6g–h). Penis guide slightly constricted at base, parallel-sided at middle part, then tapering gradually to blunt apex in ventral view (Fig. 6g). Parameres stout, tapering toward apex, distinctly shorter than penis guide, densely covered with long setae at apices and inner sides (Fig. 6h).

Female unknown.

##### Type material.

**Holotype**: male, “Wenfeng Temple, Lijiang, Yunnan, (26°48.64'N, 100°12.15'E), 20. IV. 1997, Yao DF *et al*. leg (handwritten) / Scymnus (Pullus) robustibasalis Yu, sp. n. (printed, red label)” (BAAF). **Paratype**: 1♂, “Wenfeng Temple, Wenbishan, Lijiang, Yunnan, (26°48.64'N, 100°12.15'E), 20. IV. 1997, Yao DF *et al*. leg (handwritten) / 970512-2 (handwritten) / Paratype (printed, yellow label), Scymnus (Pullus) robustibasalis Yu, sp. n. (printed)” (BAAF).

##### Other material examined.

**Sichuan**: 1♂, Laba River National Nature Reserve, Tianquan, 30°0.58'N, 102°27.59'E, ca 1100 m, 4. X. 2007, Chen XS leg. **Yunnan**: 1♂, Hutiaoxia, Lijiang, 27°10.97'N, 100°3.16'E, ca 1100 m, 3. IX. 2005, Qin ZQ leg.

##### Distribution.

China (Sichuan, Yunnan).

#### Scymnus (Orthoscymnus) duomaculatus

Taxon classificationAnimaliaColeopteraCoccinellidae

Chen & Ren sp. n.

http://zoobank.org/D56A3761-3318-4CC9-BBC3-FE4C77B2FFA1

Figs 7a–h , 9

##### Diagnosis.

This species closely resembles Scymnus (Orthoscymnus) robustibasalis in having black pronotum but can be distinguished from it by the black elytra with two brown spots (Fig. 7a) and parameres expanding toward their rounded apices, slightly longer than penis guide in lateral view (Fig. 7h). It is also similar to Scymnus (Pullus) rufomaculatus Canepari, 2012 in dorsal colour pattern, but can be separated from it by the stout penis guide in lateral view (Fig. 7h) and the different shape of penis capsule (Fig. 7e).

##### Description.

TL: 1.62–1.83 mm, TW: 1.12–1.24 mm, TH: 0.71–0.81 mm, TL/TW: 1.44–1.47, PL/PW: 0.51–0.53, EL/EW: 1.24–1.25, HW/PW: 0.57–0.60, PW/EW: 0.71–0.74.

Body oval, moderately convex, dorsum covered with white pubescence (Fig. 7a–c). Head black. Antennae and mouthparts dark brown. Pronotum and scutellum black. Elytra black with two kidney-shaped brown spots near suture. Underside entirely black. Legs dark brown.

Head with fine frontal punctures, as large as eye facets, 0.5–1.0 diameter apart. Eyes densely faceted, interocular distance 0.5 times head width. Pronotal punctures larger than those on frons, 1.0–2.0 diameters apart. Surface of elytra with punctures larger than those on pronotum, separated by 1.0–2.0 diameters. Prosternal process trapezoidal, 2 times as long as its width at base; with lateral carinae extending to anterior margin of prosternum, distinctly convergent anteriorly. Abdominal postcoxal lines extending nearly to posterior margins of abdominal ventrite 1 (Fig. 7d), area enclosed by lines finely punctate, broadly smooth along line. Abdominal ventrite 5 truncate and ventrite 6 strongly emarginated apically in male.

Male genitalia. Penis slender (Fig. 7e); penis capsule sclerotized with small inner arm and large outer arm; apex of penis slightly sinuated with membranous appendage (Fig. 7f). Tegmen stout (Fig. 7g–h) with penis guide parallel-sided from base to 3/4 length, then tapering gradually to blunt apex in ventral view (Fig. 7g). Parameres curved at base, expanded toward apex, slightly longer than penis guide, sparsely covered with long setae at apices (Fig. 7h).

Female externally similar to male but with abdominal ventrites 5 and 6 rounded apically.

##### Type material.

**Holotype**: male, No. SCAU (E) 13197, **China: Tibet**: Jilong Town, Jilong County, 28°23.00'N, 85°19.60'E, ca 2900 m, 29. X. 2011, Li WJ leg. **Paratypes (9): Tibet**: 2♀ with same data as holotype. 1♂5♀, Zhangmu Town, Nielamu County, Rikaze, 27°58.47'N, 85°58.15'E, ca 2500 m, 27. IX. 2009, Chen XS leg. 1♂, Zhangmu Port, Rikaze, 27°58.47'N, 85°58.15'E, ca 3000 m, 28. IX. 2009, Chen XS leg. (SCAU).

##### Distribution.

China (Tibet).

##### Etymology.

The species name is derived from Latin (‘*duo*-’ = two and ‘*maculatus*’ = maculate), referring to two brown spots on the elytra.

## Supplementary Material

XML Treatment for Orthoscymnus

XML Treatment for Scymnus (Orthoscymnus) smetanai

XML Treatment for Scymnus (Orthoscymnus) jilongicus

XML Treatment for Scymnus (Orthoscymnus) paradoxus

XML Treatment for Scymnus (Orthoscymnus) rhododendri

XML Treatment for Scymnus (Orthoscymnus) crispatus

XML Treatment for Scymnus (Orthoscymnus) robustibasalis

XML Treatment for Scymnus (Orthoscymnus) duomaculatus
